# The Expanding Role of Diagnostic Ultrasound in Plastic Surgery

**DOI:** 10.1097/GOX.0000000000001911

**Published:** 2018-09-05

**Authors:** Eric Swanson

**Affiliations:** Private practice, Leawood, Kans.

## Abstract

**Background:**

Ultrasound in plastic surgery is quickly finding new applications. Ultrasound surveillance may replace ineffective individual risk stratification and chemoprophylaxis for deep venous thromboses. Abdominal penetration can be a catastrophic complication of liposuction. Preoperative screening for fascial defects may reduce risk. Limiting buttock fat injections to the subcutaneous plane is critical for patient safety, but it is difficult to know one’s injection plane.

**Methods:**

The author’s use of diagnostic ultrasound was evaluated from May 2017 to May 2018. Ultrasound scans were used routinely to detect deep venous thromboses. Patients undergoing abdominal liposuction and/or abdominoplasty were scanned for possible hernias. Other common applications included the evaluation of breast implants, breast masses, and seroma management. The device was used in surgery in 3 patients to assess the plane of buttock fat injection.

**Results:**

One thousand ultrasound scans were performed during the 1-year study period. A distal deep venous thrombosis was detected in 2 patients. In both cases, the thrombosis resolved within 1 month, confirmed by follow-up ultrasound scans. A lateral (tangential) fat injection method was shown to safely deposit fat above the gluteus maximus fascia.

**Conclusions:**

Ultrasound scans are highly accurate, noninvasive, and well-tolerated by patients. Some of these applications are likely to improve patient safety. Early detection of deep venous thromboses is possible. Unnecessary anticoagulation may be avoided. Subclinical abdominal defects may be detected. Ultrasound may be used in the office to evaluate breast implants, masses, and seromas. In surgery, this device confirms the level of buttock fat injection.

## Abstract 2

Supplemental Digital Content is available in the text.

## INTRODUCTION

Ultrasound may be broadly classified into diagnostic and therapeutic applications. Therapeutically, ultrasound has long been used for liposuction assistance in an effort to reduce tissue trauma and improve skin contraction.^1,2^

Diagnostically, ultrasound imaging has proven to be useful in reconstructive surgery for identification of perforators for a variety of flaps,^3–10^ including the anterolateral thigh flap,^3–5^ and the deep inferior epigastric perforator flap.^6,7^ Visconti et al.^11^ routinely use color Doppler ultrasound when planning lymphaticovenular anastomoses.

Ultrasound has been used to study the integrity and rotation of breast implants.^12–20^ Ultrasound is an important tool in the management of Breast Implant-Associated Anaplastic Large-Cell Lymphoma.^21^ This device is essential for the evaluation of breast masses, including those that occur after autologous fat grafting.^22^

Ultrasound has been used to quantitate changes in fat volume after fat injection of the breasts and buttocks.^23,24^ This device has also been used to measure decreases in thickness after nonsurgical fat reduction including cryolipolysis.^25–27^ Other novel applications include evaluation of facial hyaluronic acid injection and subcutaneous thickness after botulinum toxin injection.^28–30^

This tool has been used to screen patients for abdominal wall defects before liposuction or abdominoplasty.^31,32^ It has been used to evaluate repairs of the rectus abdominis diastasis, and for seroma management.^33–37^ Hand surgeons have found numerous applications, such as visualizing tendons and foreign bodies of the upper extremities and guiding injections.^36^

Intraoperative ultrasound imaging assists surgeons who perform thoracic wall, paravertebral, and transversus abdominis plane nerve blocks.^38–44^ Ultrasound guidance may be used to avoid the implant at the time of breast fat grafting,^36^ to guide iliohypogastric nerve resection in patients with chronic pain,^45^ assist in cephalic vein transposition,^46^ and to identify digital artery perforators.^47^

Two recent reviews include many of these applications.^5,48^ However, an important office application has not been widely recognized—diagnostic ultrasound for deep venous thrombosis (DVT) surveillance.^35^ The safety of buttock fat injection is a major concern because of the risk of fat embolism.^49–51^ This device may be used to evaluate the level of fat injection.^24,52^

## PATIENTS AND METHODS

A retrospective study was undertaken to evaluate the use of diagnostic ultrasound in the author’s cosmetic surgery practice over the course of 1 year, May 2017 to May 2018 (Table 1). This study was determined to be exempt by the Advarra Institutional Review Board, accredited by the Association for the Accreditation of Human Research Protection Programs, Inc.

**Table 1.**
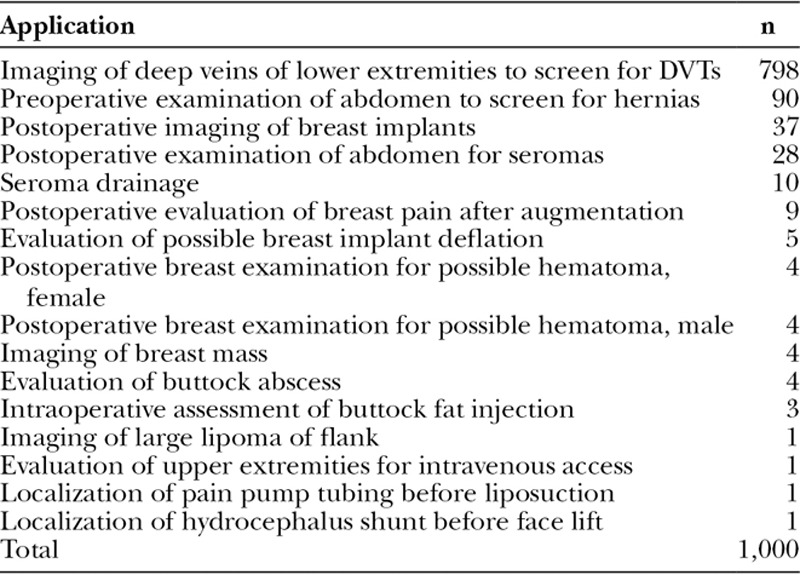
Ultrasound Examinations during May 2017 to May 2018

**Fig. 1.**
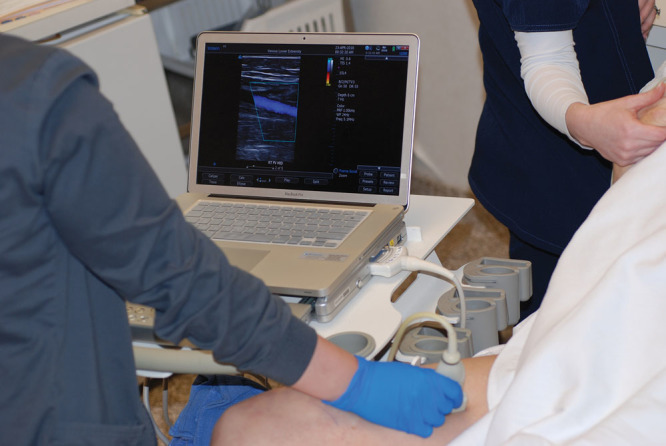
This 55-year-old woman is undergoing ultrasound imaging of her lower extremities the day after breast augmentation, liposuction, and buttock fat injection. The femoral vein appears blue on the monitor.

At the author’s clinic, Doppler ultrasound screening is offered to all plastic surgery patients undergoing surgery under total intravenous anesthesia. Scans are scheduled before surgery, the day after surgery (Fig. 1), and approximately 1 week after surgery. The Terason t3200 Ultrasound System Vascular series (Terason Ultrasound, Burlington, Mass.) is used to image the deep veins of both lower extremities, including the calf veins.^53^

This device is routinely used to image the abdomen in patients scheduled for abdominal liposuction, abdominoplasty, or the combined procedure. Ultrasound is also used to assess breast implants for the presence of folds or any other abnormality.

**Fig. 2.**
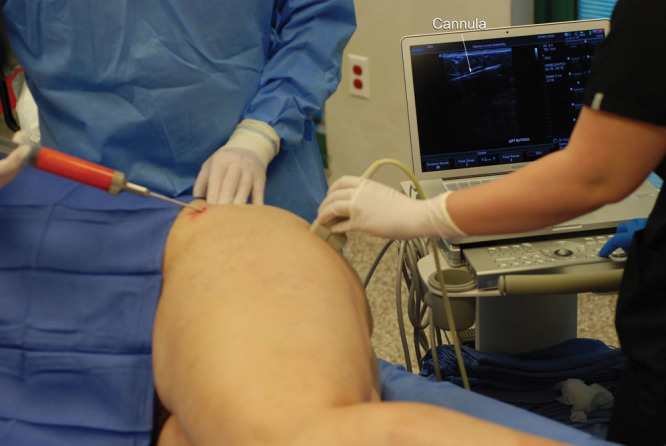
Intraoperative photograph of a 44-year-old woman undergoing fat injection of the left buttock. The monitor shows the cannula within the subcutaneous fat layer, well superficial to the muscle fascia. The video (Supplemental Digital Content 1) features the same patient.

In 3 women undergoing gluteal fat transfer, the device was used intraoperatively to visualize the level of fat injection (Fig. 2). The author prefers to inject patients in a lateral decubitus position, foregoing prone positioning, and using only 2 incisions located laterally, with no incision in the gluteal fold or intergluteal crease. This approach facilitates a tangential injection plane above the muscle fascia (**see video**, Supplemental Digital Content 1, which demonstrates intraoperative buttock fat injection with real-time ultrasound imaging of the injection plane, http://links.lww.com/PRSGO/A838).

The author does not charge patients or insurance companies for any of these uses. The cost is absorbed by the author’s practice.

## RESULTS

**Fig. 3.**
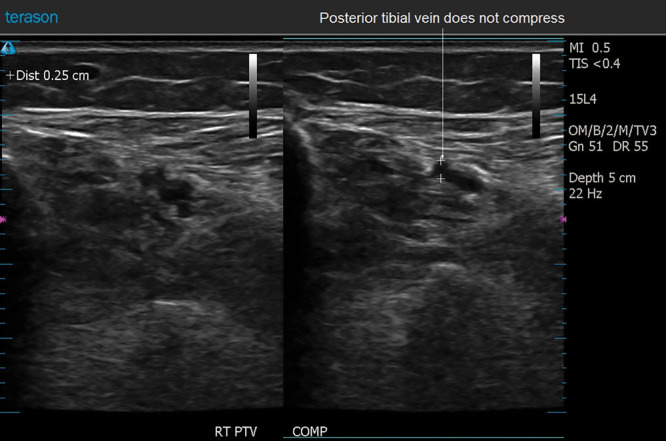
This 49-year-old woman returned in follow-up 6 days after surgery. Her ultrasound scan demonstrated a thrombus in a right posterior tibial vein. There was no evidence of popliteal extension. This image shows noncompression of one of the posterior tibial veins, indicating the presence of an intraluminal mass. The patient’s color flow and waveform images are shown in Figures 4, 5.

**Fig. 4.**
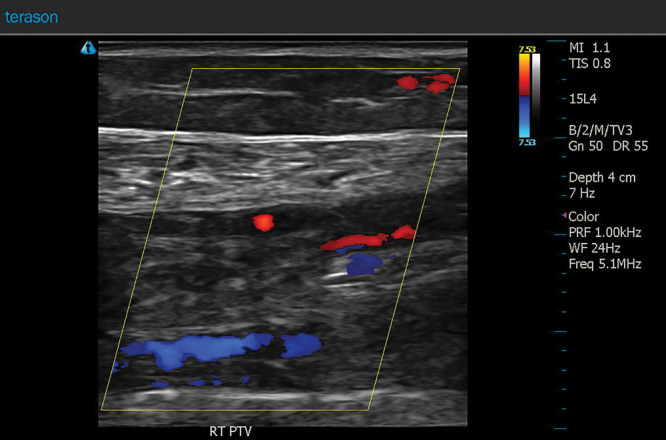
A longitudinal color flow Doppler ultrasound image shows diminished flow in the right posterior tibial vein.

**Fig. 5.**
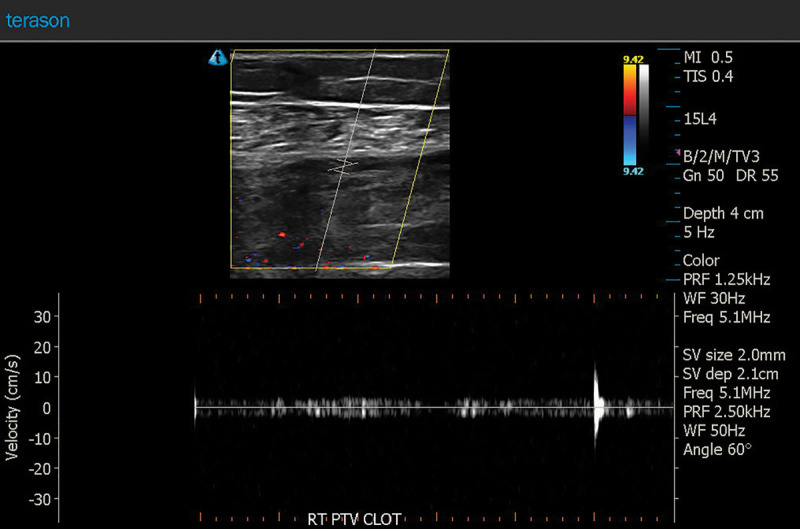
Waveform analysis shows absent blood flow in the right posterior tibial vein.

The most common application was for DVT surveillance (Table 1). During the 1-year study period, 2 DVTs were detected. Figures 3–5 depict ultrasound images of a 49-year-old woman 6 days after a face lift. Ultrasound surveillance detected an asymptomatic thrombosis of the right posterior tibial vein. She was treated with apixaban (10 mg p.o. bid for the first week, then 5 mg p.o. bid). The other affected patient was a 39-year-old woman who complained of a painful right ankle 1 week after an abdominoplasty. An ultrasound scan detected a distal thrombosis of a right posterior tibial vein. Both patients were monitored with weekly ultrasound scans, and the thromboses completely resolved within 1 month. Surprisingly, the second patient with the symptomatic thrombosis elected not to fill her prescription for rivaroxaban, against medical advice. Her thrombosis, and her symptoms, resolved spontaneously.

Other applications included seroma management, evaluation of breast implants, detection of possible hematomas, intraoperative evaluation of fat injection (Fig. 2), and imaging breast masses (Table 1).

## DISCUSSION

Diagnostic ultrasound is finding a large and important number of applications in plastic surgery that can lead to transformative improvements in patient care. Of the 47 publications on plastic surgical applications of ultrasound enumerated in the introduction,^3–48,52^ only 3 studies^25,38,42^ were published before 2012. A recent review was boldly titled “Plastic Surgeon-Led Ultrasound.”^36^ Indeed, plastic surgeons are at the forefront of these novel applications.

The value of “point of care” diagnosis has been recognized.^36,54^ Making the diagnosis in the plastic surgery office expedites patient treatment and reduces the inconvenience and expense of a patient visit to a hospital radiology department.^36^ Courses are now being offered to familiarize physicians with ultrasound use.^54^ Sonograms for DVT evaluation are ideally conducted by trained sonographers who are credentialed in vascular studies. The author does not perform any ultrasound examinations personally.

### Surveillance for DVT

DVT is a serious surgical complication that can lead to fatal pulmonary embolism.^55^ To reduce the frequency of this postoperative condition, prophylactic anticoagulation (ie, chemoprophylaxis) has been recommended for patients deemed to be at high risk.^56–59^ The author has challenged the efficacy and safety of chemoprophylaxis.^60–64^ Despite efforts to accurately predict which patients will develop a DVT after surgery,^57,59^ this goal remains elusive.^60–64^

Clinical diagnosis of venous thromboembolism (VTE) is known to be unreliable.^65–72^ A clinical diagnosis is confirmed by ultrasound or venography in only about 20–35% of patients,^66,67,69,72^ making objective confirmation mandatory.^66^ When compression ultrasound is complemented by Doppler color flow evaluation (“duplex” sonography), the sensitivity for thrombosis detection is about 96%, with a high negative predictive value (99%).^73^

Patients whose DVTs are detected by ultrasound may be followed with weekly sonograms to document resolution.^61^ Those patients presenting with distal thromboses may be treated as outpatients and prescribed an oral anticoagulant, such as rivaroxaban or apixaban, reducing the need for injectable enoxaparin. A complete ultrasound screening examination of both lower extremities, including the calf veins, takes about 20 minutes for an experienced sonographer.^74^ Deep venous thromboses developing within the first week after surgery in plastic surgery patients tend to be limited to the calf veins.^74,75^

The cost of the system used by the author is about $30,000, including a 5-year warranty, or $6,000 per year. The cost of employing part-time sonographers over the course of a year is about $20,000, which is similar to the cost of a single hospitalization for the treatment of a DVT.^76^ The author employs a full-time sonographer at a cost of about $40,000 annually. Such an effective “early warning system” compares favorably to the cost of many other plastic surgery devices in the marketplace. Any plastic surgeon who has encountered a patient death from a pulmonary embolism understands the enormity of this complication, not just financially but emotionally.^53^ Hematomas are distressing to patients and surgeons; any method that mitigates this risk is welcome, quite aside from the extra cost of managing this complication.^53^

Patients are grateful to know that their surgeon emphasizes safety^35^ and is willing to provide an important additional safety measure at no extra cost. Open discussions with patients regarding the risk of VTE and methods to reduce risk are helpful. Consulting physicians are often impressed with this heightened level of concern. Such safety measures are likely to reduce our shared medicolegal liability.^53^

Some investigators question whether knowledge of a thrombosis is even desirable, arguing that a distal thrombosis does not require treatment. It is true that most distal thromboses are likely to spontaneously resolve,^77^ and this phenomenon was demonstrated by 1 of the 2 affected patients treated within the study period. However, thromboses may also propagate. A prudent course of management, and one supported by the American College of Chest Physicians,^78^ is weekly ultrasound scans to confirm resolution.^61^

Ultrasound screening avoids unnecessary anticoagulation and identifies patients with early subclinical thromboses. One need not wait for a large proximal thrombosis to propagate unseen and undetected. As proponents of chemoprophylaxis point out, the presenting clinical sign of VTE may be sudden death.^79^

### Preoperative Screening for Abdominal Defects

In addition to early detection of DVTs, ultrasound screening may also help to prevent another rare but devastating complication—visceral perforation.^29^ Ultrasound evaluation is particularly important in patients with previous abdominal surgery and scarring. In the author’s practice, all patients undergoing liposuction and abdominoplasty are screened preoperatively using ultrasound.

### Intraoperative Use

Oni et al.^36^ use ultrasound to visualize the pectoralis muscle, ribs, and lungs to guide breast fat injection and avoid pleural penetration. Salviz et al.^43^ report that adding ultrasound-guided thoracic paravertebral blocks to general anesthesia reduces analgesic consumption in breast reduction patients. Ultrasound guidance helps to select needle insertion sites, provide depth information, improve the accuracy of the block, and minimize the risk of pleural puncture.^43^

### Evaluation of Gluteal Fat Injection

This risk of fat embolism at the time of buttock fat transfer has received much attention recently in the plastic surgery literature. This catastrophic complication is caused by a tear in one of the large gluteal veins and fat embolism to the heart and lungs.^49–51^ Alarmingly, cadaveric dissections show that even superficial fat injection into the gluteus maximus muscle leads to fat (or rather its surrogate, apple sauce) accumulation around the deep gluteal veins, because there is no deep muscle fascia to act as a barrier.^51^ Subcutaneous fat injection is recommended.^49–51^ However, it is difficult for surgeons to know their plane of injection.^49^ Intraoperative ultrasound (Supplemental Digital Content 1) provides a means to check one’s method to be sure the fat is injected in the desired subcutaneous plane. Intraoperative ultrasound is not used routinely.

### Postoperative Uses of Ultrasound

**Fig. 6.**
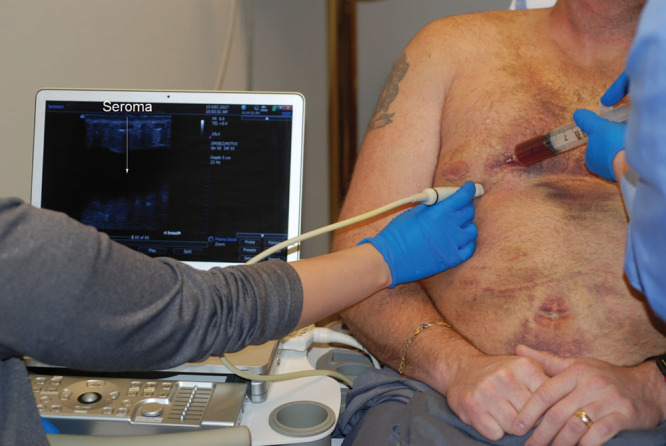
This 42-year-old man underwent liposuction of the abdomen, flanks, and breasts, and bilateral subcutaneous mastectomies for gynecomastia. He is seen 8 days after surgery. An ultrasound scan of his lower extremities was negative. However, a scan of his breasts revealed seromas. Under ultrasound guidance, the right breast was aspirated for a total of 100 cc of fluid. A volume of 80 cc was obtained from the left breast. The patient required 3 additional aspirations over the next week.

Other useful clinical applications of diagnostic ultrasound include diagnosing and treating seromas (Fig. 6). Swelling of the lower abdomen is common after abdominoplasty. Although fluctuance is a clear sign of a fluid collection, it may be difficult to differentiate a small fluid collection from postoperative edema. Sometimes patients report a popping sensation after abdominoplasty, possibly indicating that a suture has loosened. The rectus abdominis muscles may be imaged, confirming that the repair is intact, which is reassuring to patients. Abdominoplasty patients may have nerve-related abdominal pain. An ultrasound scan in this situation can be reassuring to the patient, who may not be easily convinced that nothing is wrong based on clinical examination alone.

An evolving postoperative hematoma may be difficult to distinguish from swelling or simply a high implant position. The surgeon may be in surgery and unable to immediately examine a patient in the recovery room. An ultrasound examination makes the diagnosis with high reliability. Definitive arrangements may be made for the patient’s return to the operating room or discharge, without waiting to see if the degree of swelling changes.

Coleman et al.^25^ used ultrasound to evaluate the fat layer thickness after cryolipolysis. Recently, Adjadj et al.^27^ used ultrasound to measure the decrease in fat thickness after cryolipolysis. Ultrasound imaging can quantitate changes in buttock thickness after fat transfer.^24^ This method is more sensitive than magnetic resonance imaging for detecting oily cysts.^23^

### Breast Implant Evaluation

Although magnetic resonance imaging has been considered the gold standard for breast implant rupture detection,^14^ ultrasound imaging is the preferred initial investigation in Europe.^18^ Sisti et al.^18^ report an 87% concordance between ultrasound and magnetic resonance imaging, and a close correlation between imaging signs and findings at explantation. Bengtson and Eaves^12^ report that surgeon-performed high-resolution ultrasound accurately identified the implant status and correlated well with radiologist-performed ultrasound, magnetic resonance imaging, and surgical findings. The greater affordability, availability, and the dynamic real-time visualization provided by ultrasound are advantages in both the screening and diagnosis of breast implant shell failure.^12^ Sieber et al.^20^ used ultrasound to evaluate postoperative rotation of shaped breast implants, finding that this phenomenon is much more common than previously thought, occurring in 42% of patients.

It is not unusual for patients to return in follow-up complaining of breast pain. Usually there is no history of a specific injury after surgery. Clinical examination is typically unremarkable. The surgeon reassures the patient that this pain is likely caused by a tear in the capsule. An ultrasound scan in the office shows an intact implant. This examination, which the patient can view herself, helps to relieve her apprehension that there may be another cause for the pain. Women may return with a concern regarding a palpable breast irregularity. In thin patients, a fold may be palpated, visible on the ultrasound scan. Implant deflation may be confirmed.

### Evaluation of Breast Masses

A superficial mass may be imaged to determine whether it is cystic or nodular. Cystic lesions are typically benign and may require no further investigation. Nodular lesions are referred for additional radiographic workup at a hospital or radiology clinic, possibly leading to a biopsy.

The initial investigation of an enlarged breast should include ultrasound evaluation specifically for a fluid collection, a breast mass, or enlarged regional lymph nodes.^21^ Ultrasound guidance helps to protect the breast implant and guide fine needle aspiration, and may be performed in the clinic setting.^21^

### Breast Implant-associated Anaplastic Large-cell Lymphoma

Adrada et al.^80^ reviewed 44 BIA-ALCL patients with imaging studies and reported on the sensitivity and specificity for detecting an effusion using ultrasound (84% and 75%, respectively), computed tomography (55% and 83%), magnetic resonance imaging (82% and 33%), and positron emission tomography/computed tomography (38% and 83%). The authors recommend ultrasound as a screening tool, and reserve positron emission tomography/computed tomography as part of the oncologic workup.

### Miscellaneous Uses

This tool is also useful for imaging large soft-tissue masses to be sure there is no deep extension. This study has limitations. It represents an early experience of a single surgeon. No doubt many other uses of this technology will become apparent in the near future.

## CONCLUSIONS

Ultrasound technology is widely applicable to plastic surgery. Sonograms are highly accurate, noninvasive, and well-tolerated by patients. Diagnoses are expedited, improving patient safety. Early detection of DVTs is possible. Subclinical abdominal defects may be visualized. Ultrasound may be used in the office to evaluate breast implants, masses, and fluid collections. In surgery, this device confirms the level of buttock fat injection.

**Video Graphic 1.**
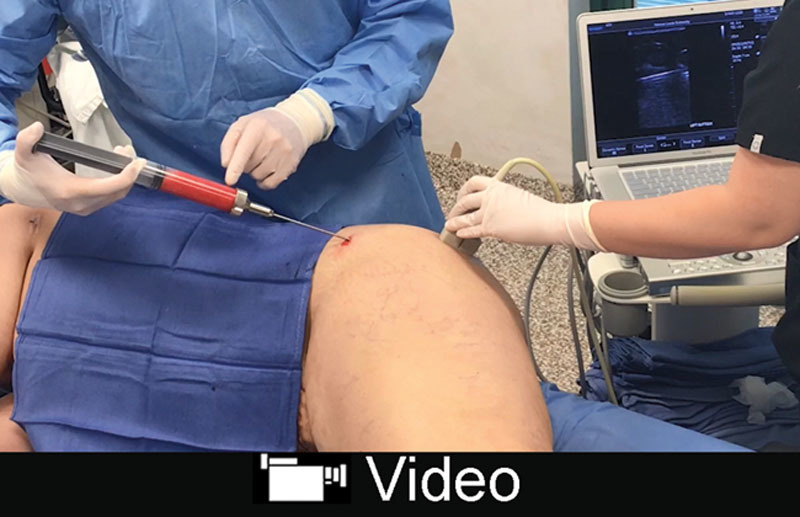
See video, Supplemental Digital Content 1, which displays an intraoperative video of a 44-year-old woman undergoing fat injection of the left buttock. The patient is positioned on her right side. Fat harvesting has already been completed. The patient had liposuction and an abdominoplasty. The monitor shows the cannula within the subcutaneous fat layer. Fat can be seen exiting the cannula (red circle), well above the gluteus maximus muscle fascia. The 2-second ultrasound imaging segment is shown at normal speed, but repeated ×7 to allow the reader enough time to view the fat escaping from the end of the cannula, http://links.lww.com/PRSGO/A838.

## ACKNOWLEDGMENTS

The author thanks Christina Engel, RT, for performing sonograms and for data collection.

## Supplementary Material
